# Gut Microbiota Dysbiosis and Sleep Disorders: Culprit in Cardiovascular Diseases

**DOI:** 10.3390/jcm13113254

**Published:** 2024-05-31

**Authors:** Barbara Pala, Laura Pennazzi, Giulia Nardoianni, Federica Fogacci, Arrigo F. G. Cicero, Laura Di Renzo, Emanuele Barbato, Giuliano Tocci

**Affiliations:** 1 Division of Cardiology, Department of Clinical and Molecular Medicine, University of Rome Sapienza, Sant’Andrea Hospital, 00189 Rome, Italygiulia.nardoianni@uniroma1.it (G.N.); emanuele.barbato@uniroma1.it (E.B.); 2 Department of Obstetric Sciences, Faculty of Medicine and Surgery, Catholic University Sacro Cuore, 00168 Rome, Italy; 3 Hypertension and Cardiovascular Risk Research Group, Medical and Surgical Sciences Department, University of Bologna, Sant’Orsola-Malpighi Hospital, 4013 Bologna, Italyarrigo.cicero@unibo.it (A.F.G.C.); 4 Cardiovascular Medicine Unit, IRCCS AOUBO, 40138 Bologna, Italy; 5 Section of Clinical Nutrition and Nutrigenomic, Department of Biomedicine and Prevention, University of Rome Tor Vergata, Via Montpellier 1, 00133 Rome, Italy; laura.di.renzo@uniroma2.it; 6 School of Specialization in Food Science, University of Rome Tor Vergata, 00133 Rome, Italy

**Keywords:** cardiometabolic risk, cardiovascular risk, gut microbiome, sleep disorders, systematic review, PRISMA

## Abstract

**Background:** Over the past decade, the gut microbiome (GM) has progressively demonstrated to have a central role in human metabolism, immunity, and cardiometabolic risk. Likewise, sleep disorders showed an impact on individual health and cardiometabolic risk. Recent studies seem to suggest multi-directional relations among GM, diet, sleep, and cardiometabolic risk, though specific interactions are not fully elucidated. We conducted a systematic review to synthesize the currently available evidence on the potential interactions between sleep and GM and their possible implications on cardiometabolic risk. **Methods:** A systematic review was conducted following the Preferred Reporting Items for Systematic Reviews and Meta-Analyses (PRISMA) statement for reporting systematic reviews and meta-analyses, including articles from January 2016 until November 2022. Narrative syntheses were employed to describe the results. **Results:** A total of 8 studies were selected according to these criteria. Our findings indicated that the sleep disorder and/or the acute circadian rhythm disturbance caused by sleep–wake shifts affected the human GM, mainly throughout microbial functionality. **Conclusions:** Sleep disorders should be viewed as cardiovascular risk factors and targeted for preventive intervention. More research and well-designed studies are needed to completely assess the role of sleep deprivation in the multi-directional relationship between GM and cardiometabolic risk.

## 1. Introduction

Despite significant efforts to reduce the atherosclerotic cardiovascular disease (ASCVD) burden through conventional risk factor control, cardiovascular diseases (CVDs) remain the leading cause of morbidity and mortality worldwide [1]. The prevalence of obesity, type 2 diabetes mellitus (T2DM), and metabolic syndrome (MetS) continues to rise and is now considered the largest non-infectious pandemic, leading to a further increase in ASCVDs [2]. Regrettably, there is a significant challenge in improving adherence to lifestyle advice, which poses a considerable obstacle to preventive efforts aimed at reducing the burden of ASCVDs at both individual and population levels [3]. Chronic exposure to environmental stressors, such as poor diet quality, sedentary behavior, sleep deprivation, and psychosocial stress, affects various pathways related to ASCVD development and progression, including body composition, cardiorespiratory fitness, muscle strength and functionality, and the intestinal gut microbiome (GM) [2].

Healthy sleep, characterized by adequate duration, good quality, appropriate timing, and the absence of sleep disorder, is increasingly recognized as a critical component of cognitive, emotional, and physical health [4]. However, while we acknowledge the importance of diet and exercise in reducing the risk of CVD and cardiometabolic diseases, sleep is not often clinically considered a modifiable risk factor [5]. Indeed, to promote optimal health and well-being, the American Academy of Sleep Medicine and the Sleep Research Society recommend that adults between 18 and 60 years of age obtain 7–9 h of sleep per night [6]. However, nearly 50% of individuals do not comply with this advice, with 35% of the US adult population (equivalent to 80 million people) sleeping for 6 h or less [7].

Over the past half-century, sleep habits have undergone significant changes. Factors such as increased artificial lighting, shift and night work schedules, the availability of 24 h services, and the widespread use of electronic entertainment and communication technology are believed to contribute to the modification of habitual sleep duration [8,9]. Disruptions in sleep and sleep/wake cycle have been associated with both short-term consequences, such as increased stress responsivity and psychosocial issues, as well as long-term health effects, including CVDs and cancer [10]. Epidemiological and community-based data strongly suggest that short sleep duration may be a risk factor for CVDs, particularly hypertension. Individuals sleeping for 6 h or less have a 13–31% higher risk of developing future hypertension compared to those sleeping for 7–8 h [8]. To delve into specifics, recent research by Li, J. et al. [11], using a dose–response analysis, demonstrated that a reduction of 1 h of sleep per 24 h is associated with a 3–11% increased risk of all-cause mortality and cardiometabolic consequences such as CVD and T2DM. Furthermore, prolonged sleep deprivation triggers various mechanisms associated with CVDs, including endothelial dysfunction characterized by increased vascular inflammation and production of reactive oxygen species, as well as sympathetic upregulation, even in healthy young adults [8].

Recent studies linked dysregulated GM to an increased risk of T2DM, obesity, and other cardiometabolic diseases, particularly in individuals experiencing chronic sleep deprivation [12]. Considering the significant role of GM in sleep disorder-related pathologies [13,14], the potential implication of sleep quality on GM has garnered growing interest. Emerging evidence suggests a bidirectional relationship between sleep quality and GM, facilitated by the brain–gut microbiome axis (BGMA), which serves as a communication channel between the brain and the gut [15,16]. Multiple factors, influenced by the BGMA, may contribute to sleep disorders, mainly including the vagus nerve, but also the immune system, the neuroendocrine system, and bacterial metabolites such as short-chain fatty acids (SCFAs), trimethylamine-N-oxide (TMAO), bile acids, and polyphenols, which, when dysregulated, have the potential to contribute to CVD [17].

Furthermore, the GM plays a role in the production of serotonin precursors and essential signaling molecules for the central nervous and neuroendocrine systems through alterations in the tryptophan pathway. Studies have shown that germ-free animals with low levels of circulating tryptophan can restore normal levels with the colonization of a healthy microbiota, indicating the dependence of tryptophan levels on the microbiota [18]. Additionally, serotonin, a precursor of melatonin, which regulates the sleep–wake cycle, has been found to be directly correlated with certain intestinal bacteria [19]. However, there is insufficient evidence to directly prove that inadequate sleep directly affects the GM [20]. 

Strong evidence indicates that sleep restriction leads to unhealthy food choices and increased energy intake, which can influence both sleep and the abundance of bacterial species in the gut [12]. Moreover, sleep restriction is associated with higher caloric intake, blood glucose levels, insulin levels, and alterations in sympathovagal balance [21]. Insomnia patients experiencing heightened inflammatory states, possibly associated with GM dysbiosis, have shown an increased risk of hypertension and cardiovascular mortality [22]. Considering that the comorbidities most often associated with sleep disorders are primarily mediated through intermittent hypoxia and sleep fragmentation [23,24], their consequences, such as systemic inflammation and free radical production, could be related to alterations and modifications in the GM [19]. Metabolic disturbances associated with sleep loss may be mediated through the overgrowth of specific gut bacteria. Conversely, the byproducts of bacterial species that thrive in response to sleep loss have the potential to induce fatigue [25]. 

Therefore, sleep quality and duration should be considered as important targets for maintaining a healthy GM composition, and the comprehensive relationship between sleep and GM should not be overlooked. To address this, we conducted a literature review to assess the impact of sleep loss on GM in healthy human adults.

## 2. Materials and Methods

### 2.1. Literature Search and Study Selection 

A systematic review was conducted following the Preferred Reporting Items for Systematic Reviews and Meta-Analyses (PRISMA) statement for reporting systematic reviews and meta-analyses [26] in Medline, Cochrane, and EMBASE databases from January 2016 until November 2022. Additionally, we searched the PROSPERO database to ensure there were no registered similar studies. PRISMA Checklist was included in the Supplementary File (Additional File S1). The study was registered in the PROSPERO database (Registration number 2023 CRD42023467539) (Additional File S2).

Literature searches were performed by using Medical Subject Heading (MeSH) terms and free text terms. We used the following search terms: “healthy adult” and “sleep quality” OR “sleep disorder” OR “sleep fragmentation” OR “sleep disturbance” OR “Sleep Wake Disorders/diet therapy” [Majr] OR “Sleep Wake Disorders/metabolism” [Majr] OR “Sleep Wake Disorders/physiopathology” [Majr] OR “Sleep Wake Disorders/prevention and control” [Majr] AND “neurotransmitter modulation” OR “vagus nerve” OR “nervous system” OR “gut-brain axis” or “Vagus Nerve/immunology” [Mesh] OR “Vagus Nerve/metabolism” [Mesh] OR “Vagus Nerve/physiopathology” [Mesh] AND “gut microbiota composition” OR “microbiome” OR “microbiota” OR “dysbiosis” OR “Gastrointestinal Microbiome/physiology”[Mesh] AND “gut microbiota modulation” or “cardiometabolic disease”. In addition, to include comprehensive data, we reviewed the references of the searched articles.

### 2.2. Eligibility Criteria

The Population, Interventions, Comparisons, Outcomes (PICO) and study design framework were followed to identify key study concepts and to facilitate the search process [27]. The use of PICO by health education specialists is becoming necessary to ensure exhaustive literature searches and to ensure that the information being investigated relates to enhancing health outcomes.

### 2.3. Population

Apparently, healthy humans, males and females, aged > 18 years, were eligible. Observational studies such as case–control, longitudinal cohort, and cross-sectional studies were included in the analysis. Articles published as conference abstracts, commentary, reviews, and case reports were excluded. Only articles with accessible full-text articles in English were included. Animal studies were excluded.

Peer-reviewed studies published in English were included if they met the following inclusion criteria: otherwise, healthy population aged > 18 years, intervention/exposure (combinations of sleep duration modification/GM analysis), and outcomes (GM modulation and/or cardiometabolic disease).

Throughout the studies, all participants were instructed to maintain their regular lifestyle without any significant changes. They did not receive any antibiotics, probiotics, prebiotics, or antifungal medication for a period of 3 months prior to the collection of samples.

In the studies included, the majority of participants were given instructions to maintain consistent dietary and activity habits before each sleep intervention. This included adhering to their usual timing for consuming breakfast, lunch, and dinner as per their previously documented regular hours.

### 2.4. Study Selection and Data Extraction

A total of 3482 records were identified, and after the removal of duplicates and initial screening, 2739 records were excluded because they did not meet the inclusion criteria (articles published as conference abstracts, commentary, reviews, meta-analyses, case reports, and main topic as focused only on cardiometabolic disease). Moreover, 200 records were excluded because they included children. Consequently, 343 abstracts were considered and a total of 28 full-text articles were assessed for eligibility: Twenty-two were excluded because they were non-observational studies, were unrelated to the predefined outcomes, had non-eligible population types, had a non-eligible control group in comparison, and had insufficient information detailed. If probiotics or paraprobiotics were administered as a daily oral supplement or other drug/specific food, the studies were excluded from the analysis. Finally, eight full-text articles were included in the review, among which five studies were performed in China, two in the USA, and one in Germany. Data were extracted, the risk of bias was assessed, and a qualitative analysis was conducted. 

**Figure 1:**
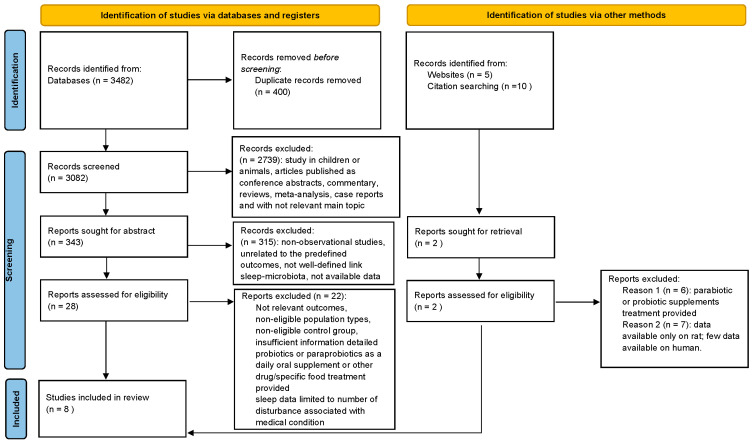
Preferred Reporting Items for Systematic Reviews and Meta-Analyses (PRISMA) flow diagram systematic reviews.

The results of the literature selection and screening process are summarized in Figure 1 (PRISMA). 

The main characteristics of selected studies are summarized in Table S1.

### 2.5. Study Quality 

A risk of bias assessment was completed for all included studies. In accordance with Evidence-Based Medicine principles, a critical analysis of a study aims to assess its internal validity, clinical relevance, and applicability. According to the Newcastle–Ottawa Quality Assessment Scale (NOS) [28], the score evaluating the study quality ranged from 5 to 8, with a mean score of 7.2 points, as reported in Table 1.

Typically, studies that achieved a score of 7 or higher on the high-quality study scale were considered, as this criterion is widely utilized due to the lack of a universally established standard for defining a high-quality study [35]. Narrative syntheses were employed to describe the results due to high levels of heterogeneity across the studies, including differences in outcomes, intervention protocols, and follow-up procedures.

## 3. Results 

A total of 8 articles were included in this systematic review, with a total of 215 participants from three different countries (North America, Asia, and Europe), mainly from China. 

Benedict, C. et al. [12] were the first to demonstrate that sleep restriction leads to deleterious effects on human GM composition, even if it was a small sample group (n = 9). They compared GM modulation after two nights of sleep restriction (sleep opportunity 02:45–07:00 h) with two nights of normal sleep (sleep opportunity 22:30–07:00 h). They reported that alpha-diversity was not altered by sleep restriction; however, the *Firmicutes* to *Bacteroidetes* (F/B) ratio was altered after sleep restriction. Also, they reported that the abundance of a few species changed after short-term sleep loss. Indeed, after sleep restriction, lower counts from the *Tenericutes* phylum and greater counts from the *Coriobacteriaceae* and *Erysipeltrichacaea* families were described. The species from these bacterial families were correlated with metabolic perturbation (liver fat content and obesity) in animal or human models [12]. 

In 2017, also Zhang et al. [32] investigated the relationship between sleep and GM. They analyzed the effects of two cycles of five nights of sleep restriction, separated by five nights of recovery sleep in 11 healthy humans. They demonstrated that the composition (beta-diversity) of the GM was resistant to sleep deprivation in humans similar to previous results [12]. 

Smit, R.P. et al. [15] described that GM diversity was positively correlated with sleep efficiency and total sleep time and was negatively correlated with sleep fragmentation. However, they determined the correlation between GM and sleep physiology over an extended period (one month). Specifically, they found that richness within the phyla *Bacteroidetes* and *Firmicutes* was positively correlated with sleep efficiency, while only the phylum *Bacteroidetes* was negatively correlated with sleep fragmentation. Moreover, the richness within the *Actinobacteria* phylum was negatively correlated with the number of awakenings. This latter evidence contrasted with Benedict, et al. [12], who found that some members of this phyla increased following sleep restriction in humans. 

Moreover, since intestinal microorganisms regulate and interact with the immune system, Smit, R.P. et al. [15] tested the correlations between interleukin 6 (IL-6), GM, and sleep. The concentration of IL-6 was positively correlated with microbiota diversity and time spent in bed and total sleep time. On the other hand, IL-6 had a neither significant positive correlation with sleep efficiency nor a negative correlation with sleep fragmentation. At the taxa level, *Corynebacterium*, which has been previously reported to have an important role in promoting sleep as a serotonin modulator and increasing the synthesis of IL-6 in some human cell types [36], was negatively correlated with a number of awakenings [15].

Recently, Whang, Z. et al. [33] investigated the impact of 40 h of sleep deprivation on GM composition and the causal role of GM in chronic inflammatory states and cognitive impairment induced by sleep disorders. They reported that sleep disorders lead to dysbiosis (reduction in alpha-diversity, modification in relative abundance), inflammatory responses (significantly increased levels of the pro-inflammatory cytokines as tumor necrosis factor-alpha, interleukin 1 beta (IL-1beta), and IL-6 and significantly decreased levels of the anti-inflammatory cytokine as interleukin 10), and cognitive impairment. In addition, sleep disorder interacts with the content of SCFAs in human fecal samples: The content of acetate, propionate, and butyrate significantly decreased. Moreover, these data were confirmed by the absence of GM inflammatory response and cognitive impairment induced by sleep disorders in germ-free mice and the reactivation of inflammatory response in germ-free mice after transplantation of a sleep-deprived microbiota. 

Liu, Z. et al. [29] simulated a sleep–wake cycle shift, one typical type of circadian rhythm disturbance in young people nowadays (without changing their sleep duration) in 22 volunteers (aged 20 to 35 years). Their results indicated that the acute circadian rhythm disturbance caused by sleep–wake shift did not induce large-scale shifts in the composition of GM; however, it affected the functional profiles of GM and interactions among them. Indeed, the relative abundances of the microbes were not significantly altered; on the other hand, the functional-profile analysis of GM revealed the purine metabolism pathways and acetyl-CoA pathway (related to SCFA metabolism) enriched.

Another very recent study by Chellappa, S.L. et al. [31] focused on the endogenous circadian system and circadian misalignment effects on human microbiota. They structured a 14-day stringently controlled circadian laboratory protocol in six healthy humans analyzing GM oral changes from their saliva samples. They confirmed the emerging evidence that the relative abundance of specific oral microbiota populations exhibited significant endogenous circadian rhythms. Indeed, circadian misalignment dramatically altered the oral microbiota landscape, such that four of five dominant phyla and eight of fourteen dominant genera exhibited significant circadian misalignment effects: Alpha-diversity analysis indicated circadian misalignment significant effect on saliva microbial communities. In addition, changes in microbial families of bacterial gut species were indicated by decreases in the relative abundance of the most prevalent phylum Firmicutes and genus *Streptococcus*, plus increases in the relative abundance of the less prevalent phylum Bacteroidetes and four reputed pro-inflammatory pathogenic oral genera within this phylum. Moreover, consistent with a previous study [29], circadian misalignment did not modify the relative abundance of oral microbiota phyla and genera; however, it significantly affected specific functional pathways associated with metabolic control and immunity: decrease in phenylalanine, tyrosine, thiamine, and tryptophan pathways, increase in the sulfur biosynthesis pathways and lipoic acid metabolism and cellular antigen pathways, which were associated with systemic inflammation. 

Two additional studies included in our review were mostly focused on microbiota and insomnia, considered as the most prevalent sleep disorder. Liu, B. et al. [30] compared the GM between insomnia patients and healthy individuals, demonstrating that the composition, diversity, and metabolic function of the GM were significantly changed. The GM alpha- and beta-diversity in insomnia patients were significantly altered. Furthermore, a decrease in the F/B ratio was also detected in the GM of insomnia group and an enrichment of gram-negative and potentially pathogenic taxa in this group with respect to controls was described. They also found that in the insomnia group, there was an increase in both vitamin B6 catabolism and folate (vitamin B9) biosynthesis, while arachidonic acid biosynthesis, which is reported to facilitate gamma-aminobutyric acid (GABA) release (which has regulatory effects on sleep functions) from nucleus striatum, was decreased.

Li, Y. et al. [34] focused on the relationship between insomnia, GM, and inflammation. Adults with acute and chronic insomnia and age/sex-matched healthy controls were recruited. Insomnia patients’ GM compared with healthy controls was characterized by lower microbial richness and diversity, depletion of anaerobes, and SCFA-producing bacteria. The chronic insomnia group had a significantly lower alpha-diversity in the microbial community. Regarding relative abundances in microbiota composition, in both insomnia group patients, decreased abundance in many anaerobic intestinal GM was reported, such as genera in *Lachnospira*, *Roseburia*, and *Prevotella 9*, with different specific abundance that correlated with the duration of insomnia. This might indicate that in sleep-deprived individuals, the microbial community may shift towards depletion of anaerobic microbes reducing SCFA production in their gut, known to have anti-inflammatory effects and to be beneficial to health. They also found a significant correlation between some specific bacteria (*Lachnospira* and *Bacteroides*) and acute insomnia disease in patients who reported poor sleep quality, but the correlation between acute insomnia disease and patients’ cytokine levels was not significant. Moreover, they [34] reported significant correlations between specific bacteria (*Faecalibacterium* and *Blautia*) with chronic insomnia, poor sleep quality, and IL-1 beta levels. 

On the basis of our analysis, sleep disturbances correspond with GM imbalances and decreased GM diversity. Additionally, specific phylum/taxa prevalence is associated with sleep disorders. Moreover, functional pathways are related to sleep disturbances, such as purine metabolism, vitamin B processes, interleukin, SCFA production, and vagus nerve activation.

## 4. Discussion

To our knowledge, this is the first systematic review focused on the effects of sleep deprivation disorders on GM in healthy humans. 

Although slight differences in GM composition related to sleep deprivation disorders have been observed, a definitive causal-effect link between these disorders and GM composition has not been established [19]. It has been suggested that the impact of severe sleep restriction on food intake and diet quality may play a role in influencing shifts in GM [20]. Furthermore, recent findings indicate that intermittent hypoxia, which is characteristic of sleep disorders, can induce systemic inflammation and free radical production. These pathogenic aspects may be associated with changes in GM composition and function. Likewise, environmental, psychological, and physical stressors that contribute to sleep disorders can have a negative impact on GM composition and function [19].

In healthy individuals, the GM is predominantly composed of the phyla Firmicutes and Bacteroidetes, which account for approximately 90% of the GM. The remaining 10% comprises phyla such as Actinobacteria, Proteobacteria, Fusobacteria, and *Verrucomicrobia*. Notably, a mucus-degrading bacterium called *Akkermansia muciniphila* has gained attention within the *Verrucomicrobia* phylum due to its influence on intestinal permeability [37]. In general, an ecosystem is considered healthy when it exhibits high biodiversity, and no single member dominates over others [19]. Benedict, C. et al. [12] were the first to provide evidence of sleep deprivation-induced changes in microbial families of bacterial gut species, which have previously been associated with metabolic pathologies. All articles included in our review took into account GM diversity, and while some studies [12,29,32] observed slight or no changes in microbial abundance and diversity, the majority reported that sleep disorders lead to gut dysbiosis and a reduction in diversity, suggesting a link between GM diversity and healthier sleep.

A critical distinction between the aforementioned studies [12,29,32] and the others [15,30,31,33,34] was that the former measured sleep over a short period and on small sample sizes, although Liu, Z. [29] attempted to increase the sample size. It is possible that short-term manipulations of sleep do not significantly influence GM diversity, and instead, microbiome diversity may be more affected in the long term. This is supported by the finding that patients with chronic insomnia exhibited significantly altered alpha-diversity of the GM, whereas the difference between the acute insomnia group and controls did not reach statistical significance [34], suggesting that longer durations of sleep disorders may have a greater impact on the patient’s microbiome diversity.

The composition of individual microbiota communities is influenced by host lifestyle and genetics. The F/B ratio is often used as an indicator of community health [37]. Some studies have found an increased F/B ratio to be associated with obesity in humans and diet or genetically induced obese mice [38,39], while other studies did not observe the same relationship [40]. Supporting these findings, an increased abundance of Firmicutes and a reduced abundance of Bacteroidetes in the GM have been observed in mice subjected to sleep fragmentation for four weeks [41]. Furthermore, these two phyla (Firmicutes and Bacteroidetes) have previously been associated with sleep quality in humans, and there is growing evidence that certain species within these phyla may modulate circadian rhythm and food intake, both of which influence sleep quality [15]. Specifically, several species belonging to the Firmicutes phylum possess genes responsible for the conversion of tryptophan to serotonin. Smith, R.P. et al. [15] have recently shown that the Corynebacterium genus is capable of synthesizing serotonin. Serotonin plays a crucial role in various functions, including being a precursor of melatonin, which regulates the sleep–wake cycle through daily endocrine fluctuations [19]. Notably, Smith, R.P. et al. [15] found a negative correlation between the abundance of this taxa and the number of awakenings during sleep [18].

Regarding the other studies included in our review, Benedict, C. et al. [12] observed an increased F/B ratio following two nights of restricted sleep compared to two nights of normal sleep. Similarly, Smith, R.P. and collaborators [15], consistent with Benedict’s findings, reported that sleep deprivation alters the F/B ratio. In contrast, Zhang, S.L. et al. [32] did not observe changes in the F/B ratio in either rat or human GM. The differences in findings between this study and Benedict’s study could be attributed to the different sample collection protocols. Benedict, C. et al. [12] considered the baseline sample to be obtained from patients’ homes, whereas Zhang, S.L. et al. [32] collected samples in a controlled laboratory environment. It is possible that the transition from home to a controlled environment could favor GM alterations within a short period. Conversely, Liu, B. et al. [30] demonstrated a decreased F/B ratio in the insomnia group compared to the control group. This discrepancy may be due to the fact that patients with insomnia did not have externally imposed restrictions on the opportunity to sleep, but they still experienced difficulties in falling asleep, staying asleep, or waking up too early, resulting in daytime impairment, which can differently affect GM dysbiosis. 

However, in order to establish a causal connection between sleep deprivation and modulation of the GM, further studies should not only focus on the taxonomic characterization of the microbiota but also employ functional and metabolic approaches to assess the microbial ecosystem. Among the studies analyzed in our review, only three considered the modulation of functional pathways.

Liu, Z. et al. [29] described alterations in purine metabolism pathways, which have also been reported in various other diseases, including inflammatory ones [42]. Additionally, they observed an enrichment in the acetyl-CoA fermentation to butanoate II pathway, which is associated with SCFA metabolism, after sleep disorders. Similarly, Chellappa, S.L. et al. [31] recently demonstrated that circadian misalignment significantly decreased phenylalanine, tyrosine, thiamine, and tryptophan pathways [43], while increasing the sulfur biosynthesis pathway, which controls the translation of specific genes and may have implications as a potential pro-carcinogen when disrupted [44]. They also observed increased abundances in lipoic acid metabolism and cellular antigen pathways, which have been associated with systemic inflammation. Additionally, Chellappa, S.L. et al. [31] provided evidence of an endogenous circadian rhythm in a mammalian system that persists even in the absence of behavioral and environmental rhythms. Emerging evidence showed that the GM may possess intrinsic circadian clocks and can be influenced by host circadian signals, such as melatonin, as well as changes in the host’s circadian rhythms due to food composition and timing [31]. To assess these functional pathways, saliva samples were used since stool analysis is not ideal due to its slow temporal dynamics, which depend on gastrointestinal motility and transit and represent an integrated measure over many hours [31].

It is worth noting that vitamin B-related pathways were significantly induced in patients with insomnia [30], resulting in vitamin B6 deficiency in the host. Vitamin B6 has been administered as a common therapeutic practice for insomnia disorder, and its deficiency can lead to fatigue and depression [45]. Furthermore, endogenously synthesized arachidonic acid facilitates the release of GABA in the striatum [46], which is essential for the serotonin pathway. Insomnia has been associated with a lower production of arachidonic acid [30].

Therefore, based on our findings, we can conclude that there is likely a correlation between stress and appetite modulation through the alteration of gut microbiota populations; indeed, the decrease in the F/B ratio and enrichment of gram-negative and potentially pathogenic taxa in insomnia patients highlight a disrupted microbial balance.

Sleep disorders contribute to gut inflammatory responses, which play a role in inflammatory processes. Wang, Z. et al. [33] reported higher levels of pro-inflammatory cytokines (including tumor necrosis factor-alpha, IL-1 beta, and IL-6) and lower levels of anti-inflammatory cytokines (including IL-10) after sleep deprivation. Moreover, their findings were supported by the absence of GM inflammatory response in germ-free mice and the presence of inflammatory response in germ-free mice after transplantation of microbiota from sleep-deprived individuals. These findings indicate that gut dysbiosis contributes to inflammatory processes. Smith, et al. [15] also investigated correlations between IL-6, intestinal microbiota, and sleep. The concentration of IL-6 was positively correlated with microbiota diversity and sleep measures, such as time spent in bed and total sleep time. Liu, Y. et al. [39] reported significant correlations between poor self-reported sleep quality in patients with chronic insomnia and their IL-1 beta levels, which was not observed in patients with acute insomnia disorder. This suggests that GM dysbiosis requires a sufficient duration of the disease to trigger inflammatory changes.

GM metabolites, such as SCFAs primarily produced through anaerobic bacterial fermentation in the large intestine, act as anti-inflammatory mediators, maintaining intestinal immune function and regulating gut barrier function [47,48]. SCFAs inhibit the production of proinflammatory mediators, including tumor necrosis factor-alpha and IL-6, in macrophages [49]. Interestingly, while some studies have found a lower abundance of SCFA-producing bacteria in obesity and type 2 diabetes, other studies have reported higher fecal SCFA concentrations in obese subjects. However, it is important to consider that dietary factors can also influence SCFA levels [12].

Five of the studies included in our review analyzed the effects of sleep deprivation on SCFA content in human feces. Two of these studies [33,34] demonstrated a significant decrease in the levels of acetate, propionate, and butyrate after sleep deprivation. Liu, Z. et al. [29], consistent with previous studies, reported an enrichment in SCFA metabolism after disturbed sleep. These results can be attributed to the reduction in the abundance of specific microbial genera following sleep deprivation, which reduces SCFA production. Additionally, sleep loss may affect the uptake and serum levels of SCFAs, thereby modifying intestinal barrier permeability, although the conclusions in this regard are not yet clear. However, Benedict, C. et al. [12] did not observe any impact on SCFA levels following sleep intervention, except for statistical trends in acetate, propionate, and total fecal SCFA levels. These differences may be attributed to the short duration of the sleep deprivation protocol.

Notably, GM is known to activate the vagus nerve, and this activation mediates the effects of GM on the brain and behavior [50]. Vagal signals from the gut can stimulate an anti-inflammatory reflex and reduce inflammation through interactions with immune cells. Further studies are needed to examine the effects of vagotomy on neuroinflammation and cognitive behavior, as well as to explore the role of the vagus nerve in the detrimental effects of sleep disorders. 

In particular, we want to underline that the gut microbiota’s influence on the autonomic nervous system (ANS), which controls heart rate and rhythm, can induce an imbalance. In cases of dysbiosis, there can be a disruption between the sympathetic and parasympathetic branches of the ANS, contributing to the development of arrhythmias [51].

To be more precise, the relationship between gut microbiota and cardiac arrhythmias involves multiple mechanisms beyond ANS regulation. Dysbiosis can lead to chronic inflammation, with elevated levels of pro-inflammatory cytokines, such as IL-6, TNF-α, and IL-1β, being linked to an increased risk of cardiac arrhythmias. Dysbiosis can also result in an overactive immune response, contributing to systemic inflammation, a known risk factor for arrhythmias [52].

Additionally, imbalances in SCFA production can affect heart rhythm by influencing inflammation and autonomic nervous system balance. High levels of TMAO are associated with an increased risk of cardiovascular diseases, including arrhythmias [53].

Moreover, dysbiosis can contribute to the development of arrhythmias by disrupting electrolyte balance, promoting oxidative stress, and compromising the integrity of the gut barrier. This leads to increased intestinal permeability (leaky gut), allowing endotoxins like lipopolysaccharides (LPS) to enter the bloodstream, promoting systemic inflammation and potentially contributing to cardiac arrhythmias [54].

Furthermore, even if our systematic review provides evidence of changes in GM abundance and diversity induced by sleep disorders, it remains an open question whether the GM can recover and how long it takes to do so after sleep deprivation is interrupted. Therefore, studies with longer intervention durations and larger sample sizes are needed to assess the effects of chronic circadian rhythm disruption on GM abundances and network organization. It is crucial to employ methodological and analytical strategies that involve well-standardized sleep interventions and taxonomic and enzymatic analysis of the GM.

### Study Limitations

The studies included in our review had some limitations that should be acknowledged. Firstly, most of the studies had small sample sizes or selective participant recruitment, with minimal adjustment for confounding factors. Thus, it is challenging to obtain reproducible results in large human populations, due to various factors such as the complexity of habitual diets, the difficulty of measuring them on a large scale, the interplay with other lifestyle variables, and the personalized nature of the GM.

Another limitation pertains to the sex distribution within the studies. This sex disparity may introduce additional variability in our findings. Furthermore, most of the studies considered in our review were conducted in Asia. It is known that regional variations in host factors exert a strong influence on GM composition. Environmental and lifestyle factors specific to certain regions could potentially impact the participants’ GM and confound the study outcomes. To limit potential heterogeneity among studies, we excluded studies involving people diagnosed with obstructive sleep apnea. To strengthen our conclusions, multicenter studies involving participants from diverse regions of the world would be necessary.

Another limitation of our study is the inability to perform a comprehensive meta-analysis to distinguish the specific effects of partial versus prolonged sleep deprivation on gut microbiota composition. The limited number of available articles and varying study designs constrained our ability to make this distinction.

Additionally, longitudinal research is needed to confirm and elucidate the relationship between changes in the GM population, alterations in temporal pathways, pro-inflammatory activation, and the pathology of sleep disorders. Long-term studies tracking participants over time would provide valuable insights into the dynamic interplay between sleep deprivation, GM composition, and inflammatory processes.

In summary, the studies considered in our review had limitations related to sample size, selective recruitment, confounding factors, sex distribution, regional variation, and the need for longitudinal research. On the other hand, our review represents the most updated, comprehensive, and systematic overview of the available evidence on the reciprocal interactions among GM, sleep, and cardiometabolic disorders. 

Even considering the intrinsic limitations of the selected studies, larger, multicenter studies and longitudinal investigations may enhance our understanding of the complex connections between GM modulation, sleep disorders, and inflammatory responses.

## 5. Conclusions

Reduced sleep duration has become increasingly prevalent and the hallmark of modern-day society with potential implications for increased incidence of cardiometabolic diseases. Sleep disorders often coexist with CVDs, deserving scientific consideration and clinical attention. Recent research suggests that GM may be susceptible to changes in different sleep patterns. It is hypothesized that a bidirectional interaction exists, whereby sleep disturbances can lead to alterations in the microbiota, and vice versa.

Our systematic review provides evidence of changes in GM abundance and diversity induced by sleep disorders. However, studies focusing not only on taxonomic characterization but also on functional modifications and the activation of various pathways would be valuable. Most of the studies reviewed agreed that sleep disorders are associated with an alteration in intestinal bacterial composition, characterized by an increase in the F/B ratio and a disruption of the intestinal barrier [15,29,30,31,33,34].

Sleep deprivation has been linked to changes in specific functional pathways [29,30,31], particularly those related to inflammation cytokine balance [15,30,33] and SCFA levels [15,29,33,34]. 

Further experiments with longer intervention periods and larger sample sizes are necessary to evaluate the effects of sleep disorders or chronic circadian rhythm disruption on GM. This will provide guidance for potential microbial therapies in clinical interventions related to sleep disorders, considering sleep as a modifiable, non-pharmacological, non-invasive therapeutic target for optimal prevention of cardiometabolic diseases. 

## Figures and Tables

**Table 1:** Newcastle–Ottawa Quality Assessment Scale of the selected studies included in the review. Methodological quality was assessed with NOS, which consists of 8 items with 3 subscales and the total maximum score of these 3 subsets is 9. Studies that scored ≥ 7 on the high-quality study scale were considered, as it is commonly used, since a standard criterion for what represents a high-quality study has not been universally established yet.

	Selection				Comparability	Exposure			Results
	Is the Case Definition Adequate	Representativeness of the Cases	Selection of Controls	Definition of Controls	Comparability of Cases ad Controls on the Basis of the Design or Analysis	Ascertainment of Exposure	Same Method of Ascertainment for Cases and CONTROLS	Non Response Rate	
Liu Z. et al. [29]	1	1	1	1	0	0	0	0	6
Liu B. et al. [30]	1	1	1	1	1	1	1	1	8
Chellappa S.L. et al. [31]	1	1	1	1	1	0	1	1	7
Zhang S.L. et al. [32]	1	1	1	1	1	1	1	1	8
Benedict C. et al. [12]	1	1	1	1	1	1	0	1	7
Smith R.P. et al. [15]	1	1	1	1	1	1	1	1	8
Wang Z. et al. [33]	1	1	1	1	1	1	1	1	8
Li Y. et al. [34]	1	1	1	1	1	0	0	0	5

## Data Availability

The original contributions presented in the study are included in the article/Supplementary Materials, further inquiries can be directed to the corresponding author.

## Supplementary Materials

The following supporting information can be downloaded at https://www.mdpi.com/article/10.3390/jcm13113254/s1, Table S1: Main characteristics and outcomes of the selected studies included in the review. Legend: SCFAs = short-chain fatty acids; GM = gut microbiota; YO = years old; CTRL = controls; IL 6 = interleukin 6; IL 1 beta = interleukin 1 beta; TNF alpha = tumor necrosis factor-alpha; AID acute insomnia disorder; CID = chronic insomnia disorder; File S1: PRISMA checklist; File S2: PROSPERO database.
