# Supplementary material for: Gut Microbiota Dysbiosis and Sleep Disorders: Culprit in Cardiovascular Diseases
Source: J Clin Med. 2024 May 31;13(11):3254. doi: 10.3390/jcm13113254 (PMC11173264; doi:10.3390/jcm13113254)
Supplement: Supplementary file 1 [file jcm-13-03254-s001.zip › additional file S2 - Prospero.pdf]

gut microbiota dysbiosis and sleep disorders: culprits in cardiovascular diseases

To enable PROSPERO to focus on COVID-19 submissions, this registration record has undergone basic automated checks for eligibility and is published exactly as submitted. PROSPERO has never provided peer review, and usual checking by the PROSPERO team does not endorse content. Therefore, automatically published records should be treated as any other PROSPERO registration. Further detail is provided here.

Citation

BARBARA PALA, Giuliano Tocci. gut microbiota dysbiosis and sleep disorders: culprits in cardiovascular diseases. PROSPERO 2023 CRD42023467539 Available from:
https://www.crd.york.ac.uk/prospero/display_record.php?ID=CRD42023467539

Review question

Is there a definitive association between sleep disorders and dysbiosis of the gut microbiota? Given the established link between gut microbiota and cardiometabolic diseases, might sleep deprivation influence the risk for cardiovascular diseases through microbiota modulation? Can sleep deprivation be considered a risk factor for cardiometabolic diseases?

Searches

Apparently healthy humans, males and females, aged >18 years, were eligible. Observational studies such as case–control, longitudinal cohort, and cross-sectional studies were included in the analysis. Articles published as conference abstracts, commentary, reviews, and case reports were excluded. Only articles with accessible full-text articles in English were included. Animal studies were excluded.

Types of study to be included

Observational studies such as case–control, longitudinal cohort, and cross-sectional studies were included in the analysis.

Condition or domain being studied

Given the established link between gut microbiota and cardiometabolic diseases, as well as the association between sleep disorders and cardiometabolic risk, we aim to investigate whether sleep deprivation can influence the risk of cardiovascular diseases via microbiota modulation.

Participants/population

Apparently healthy humans, males and females, aged >18 years, were eligible.

Intervention(s), exposure(s)

combinations of sleep duration modification/GM analysis

Comparator(s)/control

GM modulation and/or cardiometabolic disease

Context

Peer-reviewed studies published in English were included, if they met the following inclusion criteria: otherwise, healthy population aged >18 years, intervention/exposure (combinations of sleep duration modification/GM analysis), and outcomes (GM modulation and/or cardiometabolic disease).Throughout the studies, all participants were instructed to maintain their regular lifestyle without any significant changes. They did not receive any antibiotics, probiotics, prebiotics, or antifungal medication for a period of 3 months prior to the collection of samples.In the studies included, the majority of participants were given instructions to maintain consistent dietary and activity habits before each sleep intervention. This included adhering to their usual timing for consuming breakfast, lunch, and dinner as per their previously documented regular hours.

Main outcome(s)

differences in GM composition related to sleep deprivation disorders

Additional outcome(s)

increased F/B ratio; alterations in GM metabolism pathways, gut inflammatory responses after sleep deprivation

Data extraction (selection and coding)

A total of 3, 180 records were identified, duplicates will be remove; records which did not meet the inclusion criteria (articles published as conference abstracts, commentary, reviews, meta-analyses, case reports and main topic as focused only on cardiometabolic disease) will be excluded. Records which included children will be excluded. If the article are non-observational studies, are unrelated to the predefined outcomes, has non-eligible population types, has a non-eligible control group in comparison, and has insufficient information detailed will be excluded from the study

Risk of bias (quality) assessment

Newcastle-Ottawa quality assessment Scale will be used

Strategy for data synthesis

First, we analyzed differences in gut microbiota (GM) composition associated with sleep deprivation disorders. Subsequently, we will investigate the increased Firmicutes/Bacteroidetes (F/B) ratio, alterations in GM metabolic pathways, and gut inflammatory responses following sleep deprivation. Mata-analysis I think will be not possibile because there are a big difference in outcomes considered in the study

Analysis of subgroups or subsets

we did not plan this

Contact details for further information

BARBARA PALA

barbara.pala93@gmail.com

Organisational affiliation of the review

Sapienza University of Rome

Review team members and their organisational affiliations

Dr BARBARA PALA. Sapienza University of Rome

Assistant/Associate Professor Giuliano Tocci. Sapienza University of Rome

Anticipated or actual start date

01 September 2023

Anticipated completion date

26 October 2023

Funding sources/sponsors

no sponsor or founding sources

Grant number(s)

State the funder, grant or award number and the date of award

none

Conflicts of interest

Language

English

Country

Italy

Stage of review

Review Ongoing

Subject index terms status

Subject indexing assigned by CRD

Subject index terms

MeSH headings have not been applied to this record

Date of registration in PROSPERO

07 October 2023

Date of first submission

27 September 2023

Details of any existing review of the same topic by the same authors

no

Stage of review at time of this submission

Stage	Started	Completed
Preliminary searches	Yes	No
Piloting of the study selection process	Yes	No
Formal screening of search results against eligibility criteria	Yes	No
Data extraction	No	No
Risk of bias (quality) assessment	No	No
Data analysis	No	No

The record owner confirms that the information they have supplied for this submission is accurate and complete and they understand that deliberate provision of inaccurate information or omission of data may be construed as scientific misconduct.

The record owner confirms that they will update the status of the review when it is completed and will add publication details in due course.

Versions

07 October 2023

07 October 2023
