# Supplementary material for: Gut Microbiota Dysbiosis and Sleep Disorders: Culprit in Cardiovascular Diseases
Source: J Clin Med. 2024 May 31;13(11):3254. doi: 10.3390/jcm13113254 (PMC11173264; doi:10.3390/jcm13113254)
Supplement: Supplementary file 1 [file jcm-13-03254-s001.zip › jcm-3027135-supplementary.pdf]

TABLE S1

Author of the study, title and Year of publication	Country	Study	Population	Sleep disorder intervention	Sleep quality	Sample	Duration	Results on GM modifications	
			Age					Composition alteration	Functional profile modification
**Liu Z, et al.** Acute Sleep-Wake Cycle Shift Results in Community Alteration of Human Gut Microbiome. **2020**	China	Cross over study	22<br>20-35 yo	sleep-wake cycle shift: Postpone their regular sleeping time for 2 to 4 h.	N/A	Fecal samples<br>Genomic sequen cing	7 days	No significant	+ purine metabolism pathways<br>+ SCFA metabolism
**Liu B, et al.** Gut Microbiota as an Objective Measurement for Auxiliary Diagnosis of Insomnia Disorder **2019**	China	Case Control	20<br>case mean 33,00 ± 6,90<br>ctrl mean 26,10 ± 1,85	Insomnia	Polisom nography	Fecal samples<br>Genomic sequencing	Chronic exposure	GM diversity alteration Relative:<br>-FIRMICUTES<br>+BACTERIOI DES	+ folate and vitamin B related pathways<br>-arachidonic acid biosynthesis in the insomnia group
**Chellappa SL, et al.** Proof-of-principle demonstration of endogenous circadian	USA	Cross over study	6	forced Circadian desynchrony protocol with four 28-h days.	Ambulatory actigraphy	Saliva sample<br>Genomic sequencing	14 days	GM diversity alteration<br>- FIRMICUTES<br>+ pro inflamammatory Bacterioides phylum	-phenylalanine, tyrosine, thiamine and tryptophan pathways<br>+ sulfur biosynthesis pathway

system and circadian misalignment effects on human oral microbiota.<br>**2022**			18-35 yo median 27,3 ± 2,3						
**Zhang SL, et al.**<br>Human and rat gut microbiome composition is maintained following sleep restriction.<br>**2017**	USA	Cross over	11<br>29-49 yo median 37,6 ± 8,8	2 cycles of 5 nights of sleep restriction (4h), separated by 5 nights of recovery sleep 12h,	Polisomnography	Fecal sample<br><br>Genomic sequencing	14-20 day total (5 night of sleep restriction)	No differences	N/A
**Benedict C, et al.**<br>Gut microbiota and glucometabolic alterations in response to recurrent partial sleep deprivation in normal-weight young individuals.<br>**2016**	Europe	Crossov study	9<br>N/A	Sleep deprivation (sleep opportunity 02:45-07:00) and two nights of normal sleep (NS; sleep opportunity 22:30e07:00 h).	Polisonnography	Fecal sample<br><br>Genomic sequencing	4 day total (2 day og Sleep deprivation)	Relative modification:<br><br>increased F/B ratio,<br><br>higher abundances of the families Coriobacteriaceae and Erysipelotrichaceae, and lower abundance of Tenericutes	changes in fecal SCFA levels

**Li Y et al.** Gut Microbiota Changes and Their Relationship with Inflammation in Patients with Acute and Chronic Insomnia. **2020**	China	Cases and controls	96<br>20 AID<br>38 CID<br>38 ctrl<br>26-55 yo		Acute insomnia 1 week <x<3 month	Pittsburgh Sleep Quality Index (PSQI)	Fecal samples<br><br>Genomic sequencing	Chronic exposure	GM diversity alteration<br><br>depletion of anaerobes, and short- chain fatty acid (SCFA)-producing bacteria, and an expansion of potential pathobionts.<br><br>Lachnospira (decresed and Bacteroides were higher acute insomnia<br><br>Firmicutes (P=0.011) were significantly decreased in the AID group	- SCFA Levels
			Ctrl mean<br><br>38.5 ±7,0	AID mean<br><br>37,8 ±7,4						
			Ctrl mean<br><br>38.5 ±7,0	CID<br><br>43.5±6.	>3-month insomnia				Faecalibacterium and Blautia higher in chronic insomnia patients from healthy controls<br><br>Actinobacteria were over-represented in the CID	IL-1β plasma level

**Wang Z, et al.** Gut microbiota modulates the inflammatory response and cognitive impairment induced by sleep deprivation. **2021**	China	Crossover study	25<br><br>19-24 old median 22.20±0.34	40 h sleep deprivation and 8 h of recovery	Polisonnography	Fecal sample Serum and saliva sample<br><br>Western Blotting	1 week of actigraph monitoring, and 2 days in Sleep Monitoring Center: first night normal 8h sleep; then by 40 h of sleep deprivation and 8h opportunity for restorative sleep.	α-diversity of the fecal microbiome significantly decreased<br><br>Genus-level differences in the relative abundance of Pre- votella, Sutterella, Parasutterella, Fusicatenibacter, allo- prevotella, Anaeroplasma, and Elusimicrobium.	higher levels TNF-α IL-1β lipopolysaccharide and IL-6 and lower anti-inflammatory cytokine levels,<br><br>Effects of SD on the content of SCFAs in human feces:The content of acetate, propionate, and butyrate significantly decreased
**Smith RP, et al.** Gut microbiome diversity is associated with sleep physiology in humans. **2019**	USA	OS	26 adults<br><br>N/A	Sleep fragmentation not well defined , characterized by wake after sleep onset (WASO, min)	Actiwatch	Fecal and saliva sample<br><br>Genomic sequencing	1 month	richness modulation within the phyla Bacteroidetes and Firmicutes were positively correlated with sleep efficiency<br><br>Bacteroidetes was negatively corre- lated with sleep fragmentation<br><br>increased richness within the Actinobacteria contributes to high sleep quality.	SCFA producing Lachnospiraceae family, including *Blautia*, *Coprococcus* and *Oribacterium*, are negatively correlated with healthy sleep<br><br>IL-6 was positively associated with microbiome richness, with time in bed and total sleep time

**Li Y et al.** Gut Microbiota Changes and Their Relationship with Inflammation in Patients with Acute and Chronic Insomnia. **2020**	China	Cases and controls	96<br>20 AID<br>38 CID<br>38 ctrl<br>26-55 yo		Acute insomnia 1 week <x<3 month	Pittsburgh Sleep Quality Index (PSQI)	Fecal samples<br><br>Genomic sequencing	Chronic exposure	GM diversity alteration<br><br>depletion of anaerobes, and short- chain fatty acid (SCFA)-producing bacteria, and an expansion of potential pathobionts.<br><br>Lachnospira (decresed and Bacteroides were higher acute insomnia<br><br>Firmicutes (P=0.011) were significantly decreased in the AID group	- SCFA Levels
			Ctrl mean<br><br>38.5 ±7,0	AID mean<br><br>37,8 ±7,4						
			Ctrl mean<br><br>38.5 ±7,0	CID<br><br>43.5±6.	>3-month insomnia				Faecalibacterium and Blautia higher in chronic insomnia patients from healthy controls<br><br>Actinobacteria were over-represented in the CID	IL-1β plasma level
